# Next-Generation HIV-1 Therapeutics in Co-Endemic Settings

**DOI:** 10.3390/biomedicines14020330

**Published:** 2026-01-31

**Authors:** Brandon Ngo, Richard E. Sutton

**Affiliations:** Section of Infectious Diseases, Department of Internal Medicine, Yale School of Medicine, 300 Cedar St., New Haven, CT 06520, USA; brandon.ngo@yale.edu

**Keywords:** HIV-1 therapeutics, co-endemic viral infections, arboviruses, next-generation antiretrovirals, broadly neutralizing antibodies, long-acting injectable, ART, immune activation, reservoir dynamics

## Abstract

The development of next-generation HIV-1 therapeutics, including ultralong-acting antivirals, novel mechanistic classes, and curative immunotherapies, promises to overcome the limitations of lifelong, daily antiretroviral therapy (ART). However, the real-world efficacy of these treatments depends on the complex epidemiological landscapes in which they are used. In South America, HIV-1 epidemics intersect hyperendemic arboviruses, including dengue, Zika, chikungunya, and yellow fever, and regionally isolated pathogens, such as mammarenaviruses. These co-infections cause profound episodic immune activation and organ dysfunction that alter drug pharmacokinetics, disrupting healthcare access and adherence. These factors can compromise ART efficacy, promote resistance, and influence latent reservoir dynamics. This review synthesizes clinical and translational evidence of this intersection. We evaluate how emergent agents, such as capsid inhibitors (lenacapavir), long-acting injectables (cabotegravir/rilpivirine), maturation inhibitors (GSK3640254), and broadly neutralizing antibodies (bNAbs), perform in the context of co-endemic viral challenges. Specifically, we argue that therapeutic development must become “co-infection-aware” to progress toward a cure and achieve durable HIV-1 control. We provide a translational roadmap that explicitly incorporates co-infection endpoints into clinical trials, develops preclinical models that better reflect real-world viral exposures, and prioritizes implementation strategies that remain effective in the case of recurrent outbreaks. Integrating regional viral ecology into HIV-1 therapeutic research is therefore a necessary step toward developing interventions that are durable and effective on a global scale.

## 1. Introduction

### 1.1. HIV-1 Life Cycle

Since the early 1980s, nearly 90 million individuals have been infected with Human Immunodeficiency Virus type 1 or HIV-1. By 2023, there are almost 40 million people who are living with HIV-1 in the world, with two-thirds being in sub-Saharan Africa [1]. In the early 1980s, HIV-1 was described as a novel retrovirus that led to the development of acquired immunodeficiency syndrome or AIDS [2,3]. More specifically, HIV-1 belongs to the lentivirus genus of retroviruses, characterized by its irreversible integration into the host genome, ability to infect non-dividing cells, and long clinical incubation period [4,5]. The virions are encapsulated by a lipid bilayer, with the Envelope or Env mediating binding to CD4 receptors on host immune cells and fusion with the host cell membrane through glycoproteins gp120 and gp41, respectively. The matrix and capsid are also present in the virions, with viral enzymes including integrase, protease, and reverse transcriptase [6,7].

**Figure 1:**
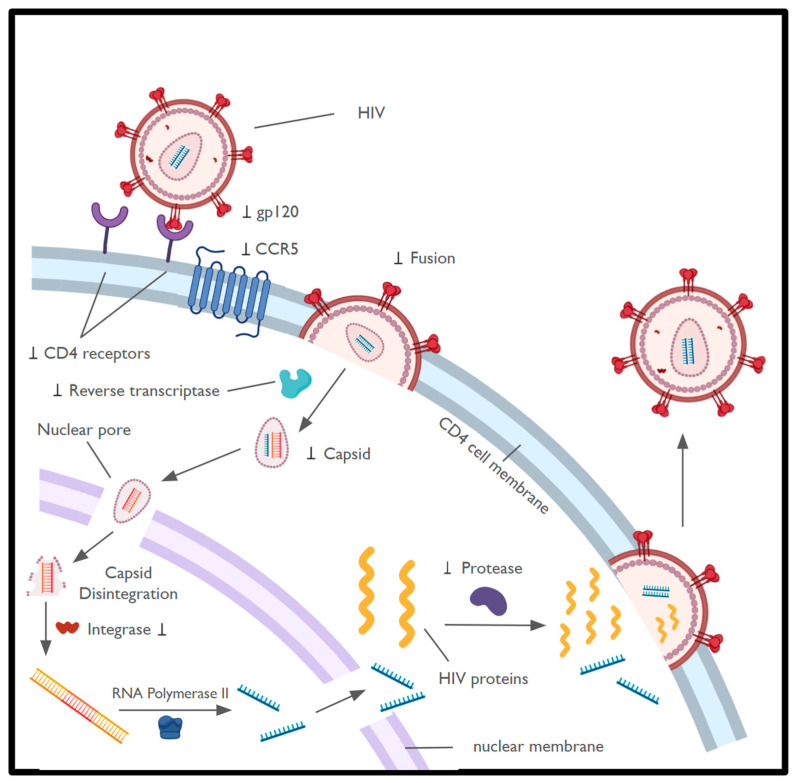
**HIV Life Cycle.** HIV-1 infects host cells through a sequence of events starting with viral attachment, continuing with viral assembly, and ending with release. The symbol ⊥ represents FDA-approved drug targets [9,14]. Single-stranded Viral RNA is shown in blue, while double-stranded viral DNA is shown in red. CD4 cell DNA is shown in yellow. Created in BioRender. Ngo, B. (2025) https://BioRender.com/vwf35g1.

The life cycle of HIV-1 begins with the virion attaching to the CD4 receptor on the surface of host immune cells, specifically CD4+ T cells and macrophages (Figure 1). After binding, Env undergoes a conformational change and binds to either CCR5 or CXCR4, which are seven trans-membrane co-receptors also present on the host cell surface. Within the gp120-gp41 complex, there are more conformational changes that allow the virus to fuse with the host cell [8,9]. Within the host cytosol and nucleus, the HIV-1 genomic RNA is transcribed into double-stranded DNA by the reverse transcriptase. The viral nucleic acid is transported into the nucleus in an intact capsid multimeric structure, which later fragments and releases the double-stranded DNA. The free double-stranded viral DNA is then integrated into the host genome [10,11]. A latent provirus may remain dormant for years until activation of the host cell. Upon activation, viral mRNA is transcribed and translated to produce viral proteins required to produce new virions [12,13].

### 1.2. HIV Therapeutics

Since the early 1990s, combination antiretroviral therapy (ART) has transformed HIV-1 seropositivity from a fatal disease to a more manageable, chronic condition by targeting various proteins responsible for HIV-1 replication [15]. However, ART is not a cure. Lifelong therapy poses adherence challenges, cumulative toxicities, and a persistent latent viral reservoir of long-lived, transcriptionally silent infected immune cells that enable viral rebound upon treatment interruption [16,17]. Next-generation therapeutics aim to address these challenges through novel antiviral mechanisms, ultralong-acting antivirals, and immune-based strategies that target the reservoir as a functional cure or long-term remission [18,19,20].

However, the success of these advanced strategies will depend on the varied global contexts with distinct clinical and immunological challenges. South America presents a critical example, harboring a significant HIV-1 presence, with complex epidemics featuring a historically dominant subtype B and a growing subtype C presence, particularly in Brazil, where, compared to a prior survey conducted a decade earlier, subtype C prevalence increased markedly, and a probable new circulating recombinant form was detected. [21,22,23]. While ART coverage is relatively high in these regions, outcomes are threatened by a parallel landscape of hyperendemic viral pathogens [1]. Regional *Aedes* mosquitoes in South America transmit dengue, Zika, chikungunya, and yellow fever viruses, causing recurrent and often overlapping epidemics affecting millions annually [24,25,26,27]. Additionally, emerging arboviruses like Oropouche and Mayaro circulate in the Amazonian regions in South America, and zoonotic mammarenaviruses like Junín and Machupo pose a persistent threat of severe hemorrhagic fever [28,29,30,31].

These endemic infections actively intersect with HIV-1 pathogenesis and care. Acute endemic viral infections induce marked systemic immune activation, characterized by widespread T-cell activation and inflammatory cytokine production [32]. Such immune activation has been linked to transient increases in HIV-1 transcription and viral “blips” [33]. Some symptoms include hepatic and hematologic dysfunction which can complicate ART pharmacokinetics and safety [34,35]. Most consequentially, they drive episodic surges in individual morbidity and healthcare utilization that can disrupt medication adherence and clinical follow-up [36,37].

This review postulates that the development and usage of next-generation HIV-1 therapeutics must consider the ecology of co-endemic viral diseases. We synthesize evidence on how South American viral infections clinically and mechanistically impact HIV-1 treatment outcomes. We then evaluate the promise and potential pitfalls of emerging antiviral agents, long-acting antivirals, biologics, and cure strategies when considering this “co-endemic environment.” Throughout this review, we explicitly distinguish between effects supported by clinical or experimental data and mechanistic hypotheses that are biologically plausible but not yet empirically demonstrated in people living with HIV-1. Finally, we propose a translational framework to ensure that innovative HIV-1 therapies are optimized for the complex environment where they are urgently needed, connecting the bridge between therapeutic benefit and public health impact.

## 2. Epidemiological Intersection: HIV-1 and Endemic Viruses in South America

South America is used here as a case study because it represents an intersection of high ART coverage, evolving HIV-1 subtype diversity, and recurrent, large-scale viral epidemics driven by arboviruses [1,22,23,27]. Importantly, the mechanisms described in this review, specifically episodic immune activation, organ-specific pharmacokinetic perturbations, and healthcare system strain, are not unique to South America [38,39,40]. Similar dynamics are present in Sub-Saharan Africa and Southeast Asia, where HIV-1 intersects with malaria, tuberculosis, viral hemorrhagic fevers, and emerging arboviruses [41,42]. Thus, insights derived from the South American context could have broad global relevance.

### 2.1. The HIV-1 Landscape in South America

South America’s HIV-1 epidemic is diverse, with an estimated 2.2 million people living with HIV-1 (PLWH) [1]. Brazil accounts for nearly half of these cases, with notable epidemics in Argentina, Colombia, Venezuela, and Peru. ART coverage is the highest among low- and middle-income regions, exceeding 80% in several countries, largely due to robust nationally funded programs [1,43]. Virologically, subtype B of HIV-1 predominates, but subtype C has recently established a significant, expanding epidemic in southern Brazil, presenting distinct therapeutic considerations [22,23,44]. The success of ART has led to an aging population of PLWH, who now face the dual challenges of long-term ART toxicities [45].

### 2.2. The Challenge of Endemic Viral Pathogens

Alongside the HIV-1 epidemic, South American is a global hotspot for other diseases, especially arboviral diseases (Table 1). Dengue is hyperendemic, with all four serotypes co-circulating and causing cyclical epidemics that strains health systems [24,46]. In this century, South America has seen the dramatic emergence of chikungunya and Zika virus, which the latter is linked to severe congenital syndromes and neurologic complications [25,27,47]. Yellow fever, though vaccine-preventable, continues to cause lethal outbreaks in sylvatic cycles, threatening peri-urban areas [26,48]. In the Amazon basin, Oropouche virus causes frequent febrile outbreaks, while the Mayaro virus emerges in urban locations [28,31,49]. Additionally, in specific rural regions of Argentina, Bolivia, and Venezuela, arenaviruses like Junín and Machupo cause Argentine and Bolivian hemorrhagic fevers, respectively, with high mortality rates [29,30,50].

### 2.3. The Co-Infection Reality

Data on the precise prevalence and outcomes of arbovirus-HIV-1 co-infection in South America remain sparse, reflecting a significant research gap. Available studies suggest that PLWH experience a similar incidence of arboviral infection as the general population, but the clinical course may be modified by immune status. Some reports find no meaningful change in short-term clinical outcomes or HIV-1 markers after Zika virus infection in well-controlled PLWH, while small case-control and series data indicate that dengue-HIV-1 coinfection can be associated with more severe dengue and longer hospital stays; however, these studies are limited by small sample sizes and single-center designs [57,58,59]. Specifically, there are limited large epidemiological studies demonstrating that arboviral endemicity alters the effectiveness of either daily or long-acting antiretroviral therapy at the population level. The virologic and immunologic consequences of these acute immune activations on HIV-1, though incompletely characterized, may be particularly relevant for long-term disease management and cure-directed strategies.

## 3. Consequences of Viral Co-Infections for HIV-1 Pathogenesis and Treatment

The interactions between HIV-1 and acute endemic viral infections are bidirectional, such that HIV-1 infection and therapy can alter the clinical course, diagnosis, and immune response to co-infecting viruses, while acute co-infections can produce immune activation, organ dysfunction, and health-system disruption that impact antiretroviral pharmacokinetics, adherence, and sustainability of HIV suppression. Below, we summarize these pathways and clarify where direct clinical evidence is abundant versus where only mechanistic or small-series data exist.

### 3.1. Immune Activation and HIV-1 Reservoir Dynamics

Acute endemic viral infections trigger a potent innate and adaptive immune response characterized by a “cytokine storm,” such as TNF-α, IL-6, and IFN-γ, and widespread T-cell activation [32,60]. This inflammatory milieu has direct implications for HIV-1. The transcription factor NF-κB, a key mediator of inflammatory signaling, is also a potent activator of the HIV-1 long terminal repeat (LTR) [61]. In people living with HIV-1 on suppressive ART, heightened immune activation has been associated with transient increases in plasma HIV-1 RNA (“viral blips”), suggesting that acute inflammatory stimuli may promote episodic HIV-1 transcription without durable viral clearance in some reports, without evidence of durable reservoir clearance at the population level [33]. This dynamic mirrors the outcome of intentional antiretroviral treatment interruption studies, a historical clinical strategy abandoned because inducing broad viral reactivation in vivo led to rapid rebound, occasional resistance, and no reduction in the latent reservoir [62,63]. In this context, acute arboviral infections may represent a naturally occurring, in vivo analog of “shock” in the absence of a corresponding “kill” mechanism. This is analogous to a study where experimentally induced HIV-1 reactivation has been shown to be insufficient for reservoir elimination without effective cytolytic immune responses [64]. Whether such transient reactivation events meaningfully expand or diversify the latent reservoir remains unknown, as direct evidence linking acute arboviral infection to durable reservoir perturbation is currently lacking.

### 3.2. Organ Dysfunction and Altered Drug Pharmacokinetics (PK) and Pharmacodynamics (PD)

Severe dengue, yellow fever, and arenaviral hemorrhagic fevers are frequently associated with hepatic injury, exhibiting elevated transaminases and acute liver failure in severe cases [27,65]. The liver is the primary site of metabolism for many antiretrovirals, particularly protease inhibitors (PIs) and non-nucleoside reverse transcriptase inhibitors (NNRTIs) via cytochrome P450 enzymes [66]. Hepatic dysfunction can lead to unpredictable drug levels and increased toxicity risk [34,67]. Additionally, severe vomiting or diarrhea can impair drug absorption, resulting in subtherapeutic concentrations of the drugs [66]. These PK disruptions during acute co-infection can lead to incomplete viral suppression that can give rise to drug-resistant HIV-1 variants, especially developing resistance against drugs with a low genetic barrier to resistance (e.g., efavirenz, rilpivirine) [21,68]. Collectively, the innate immune signaling and hepatic dysfunction pathways are well-characterized, rather than proven clinical effects, through which acute arboviral infections could intersect with HIV-1 transcriptional regulation and drug metabolism [61].

### 3.3. Healthcare Disruption and Adherence Challenges

Arboviral epidemics place immense strain on health systems as they divert resources and clinician attention [27]. For PLWH, this can mean missed clinical appointments, interrupted access to medication refills, and overwhelmed laboratories. These factors can lead to a delay in viral load monitoring [36]. At the individual level, acute febrile illness can directly reduce adherence to ART due to hospitalization, altered mental status, and the overwhelming symptoms of the co-infection. These episodic and recurrent interruptions to adherence can lead to virologic failure and accumulation of resistance, particularly for daily oral regimens like ART [37]. This vulnerability illustrates one of the strongest rationales for long-acting therapeutics in co-endemic settings.

### 3.4. Diagnostic Uncertainty and Clinical Management Complexity

The non-specific febrile illnesses of arboviruses overlap with opportunistic infections caused by HIV-1, such as tuberculosis and cryptococcosis, as well as immune reconstitution inflammatory syndrome (IRIS) [41,69]. In clinical settings where resources are constrained, misdiagnosis is common, especially if there is a lack of rapid molecular diagnostics [70]. A febrile illness in a PLWH may be incorrectly attributed to a common arbovirus, which can delay treatment for a life-threatening opportunistic infection. Conversely, assuming an illness is HIV-1-related may lead to a missed diagnosis of dengue or yellow fever, leading to poor management of the arboviral illness. This diagnostic ambiguity complicates clinical decision-making, including whether to continue or modify ART during acute illness [71].

### 3.5. Other Modifiers of Treatment Response

While co-infections are an important and underappreciated influence on HIV therapeutics, treatment response can also be determined by multiple additional factors that can interact with co-infection biology. Important modifiers include prior ART history and pre-existing resistance, timing of ART initiation and duration of suppression, regimen-specific properties and drug-drug interactions, comorbid infections, pregnancy, nutritional status, organ dysfunction, pharmacogenomic variation that modify drug variation, and health-system and socioeconomic determinants [21,72,73,74,75,76,77]. We emphasize that these factors can amplify or change the effects of co-infections. For example, pre-existing resistance or interrupted care may convert a transient PK perturbation into a loss of suppression [21]. Therefore, these modifiers must be considered alongside co-infection endpoints when designing preclinical experiments and clinical trials.

## 4. Evaluating Next-Generation HIV-1 Therapeutics Through a Co-Endemic Lens

The limitations of current ART are amplified in co-endemic settings. Next-generation strategies offer solutions, but their use in co-endemic settings must be critically assessed within this complex context (Table 2). Importantly, many next-generation HIV-1 therapeutics are intrinsically more resilient to acute illness and short-term adherence disruptions than daily oral ART; however, in co-endemic settings, their vulnerabilities shift from individual behavior to timing, pharmacokinetics, and health system reliability [21,78,79]. Distinct classes of next-generation HIV-1 therapeutics are predicted to interact differently with acute viral co-infections through class-specific mechanisms, including inflammation-driven viral replication, hepatic and hematologic dysfunction, pharmacokinetics, immune-complex formation, and health-system dependence.

### 4.1. Novel Small Molecules and Long-Acting Antivirals

Capsid Inhibitors: Lenacapavir. This therapeutic is a paradigm-shifting intervention for co-endemic regions. Characterized by its twice-yearly subcutaneous dosing, its ultralong-acting profile directly addresses the adherence disruptions that can be caused by acute febrile illnesses. A patient that receives a lenacapavir injection is protected for six months, potentially spanning an entire arboviral transmission season [80,81]. However, this benefit is dependent on a resilient healthcare system that can deliver injections precisely on schedule. The drug’s extraordinarily long pharmacokinetic tail could lead to prolonged subtherapeutic levels if an injection appointment is missed [82]. At these levels, a simultaneous inflammatory co-infection may facilitate viral replication and select for Lenacapavir resistance mutations, such as Gag M66I and Q67H [83,86]. Inadequate public-sector access further complicates rollout. For instance, although Brazil’s National Health Regulatory Agency (ANVISA) approved Lenacapavir in January 2026, regulatory approval does not itself ensure incorporation into the public health system. Pricing negotiations and procurement decisions for the Sistema Único de Saúde (SUS) are still pending, and Lenacapavir is therefore not yet available through routine public-sector HIV care [84]. Therefore, its use requires robust patient tracking and stringent delivery models.

Long-Acting Injectable ART: Cabotegravir (CAB) and Rilpivirine (RPV). The monthly or bimonthly intramuscular regimen of CAB/RPV eliminates daily pill-taking in ART, a major advantage during periods of illness or healthcare disruption [85,87]. However, real-world data highlights that virologic failure, while rare, has been associated with pre-existing NNRTI resistance and specific HIV-1 subtypes [88,89]. Acute inflammatory co-infections may further narrow the pharmacologic margin of long-acting regimens when drug concentrations approach the lower bound of efficacy, as inflammation-associated increases in viral replication can intensify selective pressure [85,86,90]. Moreover, missed injections necessitate prompt oral bridging therapy, which may be challenging to initiate during periods of individual illness or broader health system disruption [91].

Attachment and Maturation Inhibitors: Fostemsavir and GSK3640254. These agents with novel mechanisms, such as gp120 attachment and Gag cleavage inhibition, respectively, are vital for treating multi-drug-resistant HIV-1 [92,93,94]. In co-endemic settings, where adherence interruptions could lead to accumulated resistance, these drugs are crucial salvage options [95]. Their distinct resistance profiles ensure activity even when classic NRTI/NNRTI/PI options are exhausted [96]. Their role is less about the prevention of disruption but rather more about the remediation of its consequences.

Other Pipeline Agents: Islatravir, an NRTTI with extended half-life, and novel NRTIs like censavudine represent future options for infrequent dosing [97,98]. Their development should consider potential additive hematologic toxicities in the context of arboviruses known to cause thrombocytopenia.

### 4.2. Biologics and Immune-Based Strategies

Broadly Neutralizing Antibodies (bNAbs). bNAbs like VRC01 or combination regimens, such as 3BNC117 plus 10-1074, offer the potential for long-acting suppression or prevention of HIV-1, without daily drugs [99,100]. In co-endemic settings, pre-existing flavivirus immunity creates a distinct immunologic context in which broadly neutralizing antibodies (bNAbs) are administered. While no direct clinical evidence currently demonstrates altered bNAb efficacy in this setting, well-characterized Fc-mediated immune mechanisms provide a biologically plausible basis for interaction [101,102]. Acute or recent arboviral infections are associated with high circulating IgG levels, immune complex formation, and altered Fc receptor engagement, all of which could theoretically influence bNAb pharmacokinetics, tissue distribution, or clearance [103,104,105]. In addition, the phenomenon of antibody-dependent enhancement observed in flavivirus infections highlights how subneutralizing antibody-antigen interactions can modulate immune activation through Fc-dependent pathways [106]. While no direct evidence currently links flavivirus immunity to altered HIV-1 bNAb efficacy, these principles define a mechanistically grounded interaction space that warrants consideration when deploying antibody-based HIV-1 therapies in co-endemic regions [107]. On a practical level, the high cost and cold-chain requirements of current bNAb formulations are significant barriers [108]. No bNAbs are currently FDA-approved for treatment or prophylaxis of HIV-1. This pathway is already well-characterized as a mechanism, rather than a proven clinical effect, in which acute arboviral infections could intersect with antibody-mediated therapies [109].

CAR-T Cells and Gene Therapies. These approaches aim to directly target and eliminate the reservoir [110,111]. However, their complexity, cost, and requirement for sophisticated medical infrastructure places them out of reach for most health systems that grapple with dual HIV-1/arboviral epidemics [112]. Furthermore, the safety of infusing potent, activated immune cells during or following an acute inflammatory viral infection is unknown [113]. No CAR-T or gene therapeutic approaches are currently FDA-approved for treatment or prophylaxis of HIV-1.

### 4.3. Cure Strategies: “Shock and Kill” vs. “Block and Lock”

“Shock and Kill.” This approach uses latency-reversing agents (LRAs) to flush out the HIV-1 reservoir, hoping that the immune system will clear reactivated cells [114,115]. In a setting where uncontrolled, natural reactivation events are common, adding a pharmacological “shock” may be dangerous as it could potentially expand the reservoir without ensuring the effect “kill” step [116]. The already hyperactive immune state during arboviral infection could also increase the risk of toxicities from LRAs or from resultant immune activation [117].

“Block and Lock.” This alternative strategy aims to permanently silence the HIV-1 promoter and provirus, pushing the reservoir into a deep, irreversible latency [118,119]. In co-endemic regions, this approach may be more strategically sound. Rather than fighting against the frequent inflammatory “shocks,” “block and lock” agents could fortify the reservoir against them, preventing reactivation and preserving immune stability during co-infections. This makes the development of effective transcriptional silencers like didehydro-cortistatin A (dCA) particularly relevant for these settings [120]. Neither strategy is currently used to ‘flush out’ latent HIV-1 in PLWH.

## 5. A Translational Roadmap for Co-Infection-Aware Therapeutic Development

To ensure next-generation HIV-1 therapies succeed globally, research on HIV-1 next-generation therapeutics must incorporate the ecological reality of co-endemicity [121]. Within this context, distinct therapeutics, ranging from ultralong-acting agents to immune-based strategies, may interact differently with episodic immune activation, pharmacokinetic perturbations, and health-system constraints imposed by acute co-infections with diseases like arboviruses. While this translational roadmap maps biological mechanisms to potential methods to evaluate endpoints, we emphasize that there are limited, prospective population studies that directly link arboviral endemicity to HIV therapy effectiveness.

To translate the mechanistic hypotheses outlined above into actionable evidence, future studies should explicitly evaluate how acute co-infections affect HIV-1 control across therapeutic classes. Some sample study designs include cohorts in co-endemic regions with predefined sampling windows spanning baseline, including acute infection, early recovery, and convalescence, as well as population pharmacokinetic studies and early-phase clinical trials that specify co-infection–aware endpoints. Key outcomes should include plasma HIV-1 RNA dynamics, cell-associated reservoir measures, ART or biologic drug exposure, resistance emergence, and immune activation markers. Complementary preclinical models will be essential to dissect causal mechanisms, particularly for long-acting agents and antibody-based therapies.

### 5.1. Developing Physiologically Relevant Preclinical Models

Current HIV-1 therapy and cure research relies on models that do not account for periodic inflammatory insults or activations, especially from the co-endemicity of arboviruses in South America [122]. The field should prioritize:

In vitro models: Latently infected CD4+ T cells or macrophages cultures exposed to arboviral pathogens or their pathogenic components, such as viral RNA or NS1 proteins [123,124].

Animal models: Humanized mouse models, such as BLT mice, with established HIV-1 latency subsequently challenged with a relevant co-pathogen, like dengue virus, to study reservoir dynamics, immune responses, and drug efficacy under inflammatory stress [125].

### 5.2. Integrating Co-Endemic Endpoints into Clinical Trial Design

To move beyond descriptive associations, co-infection-aware endpoints must be prespecified, temporally defined, and integrated into trial protocols rather than analyzed post hoc. Trials of novel agents, especially the long-acting formulations and cure strategies, conducted in South America should involve these co-infection endpoints (Table 3):

Virologic: Magnitude and duration of HIV-1 RNA blips during acute febrile illnesses from laboratory-confirmed arbovirus infections [33].

Immunologic: Changes in immune activation markers, such as soluble (s) CD14 and IL-6, inflammation (CRP), and HIV-1-specific T-cell responses before, during, and after co-infection [126].

Pharmacologic: Population pharmacokinetics studies to model drug exposure and clearance during episodes of hepatic inflammation or other organ dysfunction [127].

Reservoir: Measurement of cell-associated HIV-1 DNA and RNA in peripheral blood before and after a documented co-infection event to assess its impact on the latent pool [128].

### 5.3. Implementation and Health System Considerations

Without resilient delivery systems, the most effective next-generation HIV-1 therapeutics may still fail. Long-acting agents require reliable appointment tracking, dosing strategies, and supply chains capable of withstanding epidemic-related disruptions. In co-endemic regions, integration of HIV-1 care with existing arboviral treatment regimens may improve outbreak anticipations and continuity of care. Cost, cold-chain requirements, and healthcare workforce limitations remain major barriers for biologics and gene-based therapies, highlighting the need for parallel investment in implementation alongside therapeutic innovation [83,129].

## 6. Conclusions and Future Perspectives

The journey towards an HIV-1 cure and optimized treatment is linked to the broader pathogenic environment. South America, with its challenge of both HIV-1 and endemic viral diseases, exemplifies the complex reality in which next-generation therapeutics must function. This situation extends globally, where co-infection exists in other regions of the world, such as Sub-Saharan Africa and Southeast Asia. Novel agents such as lenacapavir and CAB/RPV offer powerful tools to mitigate adherence challenges exacerbated by co-infections. However, their benefits are not automatic, depending on co-infection-aware deployment strategies that address pharmacokinetic risks, health system fragility, and unique immunological interactions, not to mention the cost of these therapeutics.

The research community must move beyond viewing co-infections as comorbid conditions and instead recognize them as critical modulators of HIV-1 treatments and cure outcomes. To do this, the community should focus on developing novel preclinical models, embedding relevant endpoints in clinical trials, and fostering interdisciplinary collaboration between HIV-1 specialists, virologists, and immunologists to devise a proper deployment strategy.

This narrative review synthesizes mechanistic, translational, and limited clinical literature and is not a systematic meta-analysis. Because of this, it may reflect the uneven availability of data across settings. We were generally unable to stratify findings by key modifiers, such as CD4+ T cell counts, duration or timing of ART, and prior virus resistance history, because primary studies rarely report uniform, stratified data. Likewise, other co-infections, comorbidities, pharmacogenomic variation, and health-system factors can all modify both infection course and drug performance but were beyond the scope of available evidence. We therefore present our review as hypotheses and priorities for prospective, mechanistic, and epidemiologic studies rather than conclusive statements about population-level impact.

By integrating an understanding of South America’s endemic viral landscape into the core of therapeutic development, we can ensure that the next generation of HIV-1 breakthroughs delivers not only scientific promise but also equitable and resilient care for all populations living with HIV-1. This approach will not only benefit South America but will also provide a framework for optimizing HIV-1 therapies in any region where HIV-1 intersects with other persistent infectious disease challenges.

## Figures and Tables

**Table 1:** Endemic viruses relevant to HIV therapeutics in South America.

Virus	Vector/Transmission	Typical Clinical Syndrome	Affected Organ Systems	Immunologic Features (Typical)	Relevance to HIV-1 Care	Key Citations
Dengue virus	*Aedes aegypti*; *Aedes albopictus* (mosquito)	Acute febrile illness, myalgia/arthralgia, rash; severe dengue leading to plasma leakage, hemorrhage	Liver (transaminitis, possible acute liver injury), vascular endothelium, bone marrow (thrombocytopenia)	Marked innate/adaptive activation, high cytokines (TNF-α, IL-6, IFN-γ), strong T-cell activation; frequent thrombocytopenia	Immune activation leading to transient HIV RNA “blips”; hepatic injury and thrombocytopenia can alter ART PK/toxicity and complicate management	[24,46,51,52]
Zika virus	*Aedes* spp.; sexual transmission reported	Fever, rash, conjunctivitis; congenital infection leading to fetal brain malformations	Nervous system (esp. fetal brain), occasionally liver	Acute inflammatory response; high IgG levels post-infection	Theoretical effects on antibody distribution/clearance and immune milieu relevant to antibody therapies; limited direct clinical data in PLWH	[25,27,47,53]
Chikungunya virus	*Aedes* spp. mosquitoes	High fever, severe polyarthralgia/arthritis (can persist)	Joints, possible hepatic involvement	Pronounced inflammatory cytokine responses (e.g., IL-6)	Episodic immune activation could transiently increase HIV transcription and affect adherence during disabling illness	[27,52,53]
Yellow fever virus	*Aedes* spp.; sylvatic *Haemagogus* spp.	Fever, jaundice, hemorrhage (severe disease)	Liver (often severe hepatotoxicity), kidney	Cytokine storm in severe cases; marked hepatocellular injury	Hepatic injury can cause large PK changes for CYP-metabolized ARTs and increase toxicity risk	[26,27,48,53]
Oropouche virus	Primarily *Culicoides paraensis* (biting midges); some mosquitos (by report)	Febrile illness with headache, myalgia; occasional neurological involvement	Liver, brain in rare, severe cases	Innate/inflammatory activation typical of arboviral febrile illnesses	Potential for immune activation and PK disruption during symptomatic illness; diagnostic overlap with dengue	[28,49,54]
Mayaro virus	Primary sylvatic *Haemagogus* spp.; secondary *Aedes* possible	Fever, polyarthralgia similar to chikungunya	Joints, occasional hepatic involvement reported	Cytokine-mediated inflammation	Episodic immune activation; potential diagnostic confusion with chikungunya/dengue	[31,49,55]
Junín virus	Zoonotic, rodent reservoir (*Calomys* spp.)	Argentine hemorrhagic fever: fever, hemorrhage, neurologic signs	Multi-organ including liver, vasculature	Strong innate/adaptive activation; high morbidity/mortality in severe disease	Severe hepatic/vascular injury and systemic inflammation with major implications for ART PK and clinical management	[29,30,50,56]
Machupo virus	Zoonotic, rodent reservoir (*Calomys callosus*)	Bolivian hemorrhagic fever: fever, bleeding, multi-organ dysfunction	Liver, kidney, vasculature, CNS	Intense inflammatory responses in severe disease	Severe illness can cause hepatic/renal dysfunction and disrupt ART and monitoring	[29,30,50,56]

**Table 2:** Next-generation HIV-1 therapeutics: benefits and co-endemic vulnerabilities.

Agent/Class	Mechanism of Action	Typical Dosing Interval (Published/Approved)	Key Advantage in Co-Endemic Settings	Principal Vulnerability(s) to Co-Endemic Environment	Key Citations
Lenacapavir (SUNLENCA)	Capsid inhibitor (CA)	Subcutaneous injection every 6 months (treatment regimens)	Ultra-long-acting, covers long transmission seasons; reduces reliance on daily adherence	Long PK tail, prolonged subtherapeutic exposure if injections missed; documented resistance mutations (e.g., M66I, Q67H); requires robust appointment tracking/delivery systems.	[71,72,73,74,75]
Cabotegravir and Rilpivirine (LA CAB/RPV)	INSTI + NNRTI (long-acting IM formulations)	Monthly or every 2 months (approved regimens)	Eliminates daily pill burden; helps during acute illness/health-system strain	Virologic failure associated with pre-existing RPV resistance, some subtypes, low drug exposure/missed injections; PK tail risk	[75,76,77,78,79,80,81]
Fostemsavir (Rukobia)	gp120 attachment inhibitor (temsavir active)	Daily oral (for heavily treatment-experienced)	Useful salvage option for multi-drug-resistant HIV; distinct mechanism of action	Requires oral adherence; does not prevent missed-dose problems; PK in severe hepatic illness needs monitoring	[82,83,84,85]
Maturation inhibitors (e.g., GSK3640254)	Inhibit Gag cleavage / viral maturation	Daily oral (pipeline/clinical)	Active against viruses with other class resistances; useful as salvage	Limited data in acute co-infection; requires adherence and hepatic function assessment	[84,85,86]
Islatravir (MK-8591)	NRTTI	Extended half-life (development formulations for infrequent dosing; clinical status evolving)	Potential for infrequent dosing, resulting in better adherence in unstable settings	Observed dose-dependent lymphocyte/CD4 declines in trials, hematologic safety concern where arboviruses cause cytopenias	[87,88]
bNAbs (VRC01, 3BNC117+10-1074, others)	Passive monoclonal antibodies targeting HIV Env	IV/SC dosing ranges from monthly to quarterly in trials	Long-acting suppression option without daily ART; adjunctive/remission strategy	Fc-mediated interactions and immune complex dynamics could alter PK/clearance; pre-existing flavivirus immunity is a mechanistic concern (limited direct clinical data); cost/cold-chain barriers	[89,90,91,92,93,94,95,96,97,98]
CAR-T/gene therapies	Engineered cell therapy/gene editing targeting reservoir	Single or limited infusions (complex protocols)	Potentially curative (reservoir elimination)	Highly resource-intensive; safety during/after acute systemic inflammatory infections uncertain in co-endemic, resource-limited settings	[99,100,101,102]
Shock and Kill (LRAs)	Reactivate latent HIV to enable immune/therapeutic clearance	Variable (very agent dependent)	Aimed at reservoir reduction	In frequent natural reactivation environments, pharmacologic “shock” without robust “kill” could expand/reshuffle reservoir; toxicity risk during arboviral illness	[103,104,105,106]
Block and Lock (e.g., dCA)	Deep transcriptional silencing of HIV LTR	Variable (experimental)	May protect reservoirs from frequent natural immune “shocks” and stabilize latency	Durability and off-target effects remain to be fully established	[107,108,109]

**Table 3:** Co-infection-aware clinical trial endpoints.

Endpoint Type	Specific Measure/Assay	Rationale	Key Citations
Virologic	Plasma HIV-1 RNA (copies/mL), magnitude and duration of transient blips	Directly measures transient HIV-1 transcription/reactivation during immune activation	[32]
Immunologic	sCD14, IL-6, CRP, cytokine panel, HIV-1-specific T-cell responses	Quantify immune activation that could drive HIV transcription or affect therapeutic function	[115]
Pharmacologic (PK/PD)	ART trough/peak concentrations; population PK modeling; hepatic biomarkers (AST/ALT, bilirubin)	Assess impact of hepatic/GI dysfunction on exposure and risk for subtherapeutic levels or toxicity	[116]
Reservoir	Cell-associated HIV-1 DNA and RNA	Evaluate whether acute co-infection perturbs reservoir size or transcriptional activity	[117]
Clinical/implementation	Hospitalization, adverse events, missed injections/refills, adherence metrics, viral load monitoring delays	Measure operational impact on continuity of therapy and health-system resilience	[78,118]
Exploratory/mechanistic	Antibody titers to endemic viruses, Fc-receptor profiling, immune complex measurements, sequencing for emergent resistance	Investigate mechanisms by which flavivirus immunity or immune complexes could modify bNAb PK/efficacy or select for ART resistance	[89,90,91,92,93,94,95,96,97,98]

## Data Availability

No new data were created or analyzed in this study. Data sharing is not applicable to this article.
